# Silodosin versus Tamsulosin for Medical Expulsive Therapy of Ureteral Stones: An Updated Systematic Review and Meta-Analysis of Randomized Controlled Trials

**DOI:** 10.3390/medicina58121794

**Published:** 2022-12-06

**Authors:** Hae Do Jung, Kang Su Cho, Dae Young Jun, Jae Yong Jeong, Young Joon Moon, Doo Yong Chung, Dong Hyuk Kang, Seok Cho, Joo Yong Lee

**Affiliations:** 1 Department of Urology, Inje University Ilsan Paik Hospital, Inje University College of Medicine, Goyang 10380, Republic of Korea; haedojung@paik.ac.kr (H.D.J.); seokcho@paik.ac.kr (S.C.); 2 Department of Urology, Prostate Cancer Center, Gangnam Severance Hospital, Urological Science Institute, Yonsei University College of Medicine, Seoul 06273, Republic of Korea; kscho99@yuhs.ac; 3 Department of Urology, Severance Hospital, Urological Science Institute, Yonsei University College of Medicine, Seoul 03722, Republic of Korea; dyjun881101@yuhs.ac (D.Y.J.); verban25@yuhs.ac (J.Y.J.); jjunny72@yuhs.ac (Y.J.M.); 4 Department of Urology, Inha University College of Medicine, Incheon 22212, Republic of Korea; dychung@inha.ac.kr (D.Y.C.); dhkang@inha.ac.kr (D.H.K.); 5 Center of Evidence Based Medicine, Institute of Convergence Science, Yonsei University, Seoul 03722, Republic of Korea

**Keywords:** ureteral calculi, adrenergic alpha-1 receptor antagonists, systematic review, meta-analysis

## Abstract

*Background and Objectives*: This systematic review and meta-analysis of randomized controlled trials was performed to compare the therapeutic effects and safety profiles of silodosin and tamsulosin for medical expulsive therapy (MET) of ureteral stones. *Materials and Methods:* We searched PubMed, EMBASE, the Cochrane Library, and Web of Science to identify articles published before July 2022 that described randomized controlled trials comparing silodosin and tamsulosin for MET of ureteral stones. Endpoints were stone expulsion rate, stone expulsion time, and total complication rate. *Results*: In total, 14 studies were included in our analysis. The size of ureteral stones was <1 cm. Compared with tamsulosin, silodosin resulted in a significantly higher stone expulsion rate (*p* < 0.01, odds ratio (OR) = 2.42, 95% confidence interval (CI) = 1.91 to 3.06, *I*^2^ = 0%) and significantly shorter stone expulsion time (*p* < 0.01, mean difference = −3.04, 95% CI = −4.46 to −1.63, *I*^2^ = 89%). The total complication rate did not significantly differ between silodosin and tamsulosin (*p* = 0.33, OR = 1.15, 95% CI = 0.87 to 1.52, *I*^2^ = 7%). *Conclusions:* Compared with tamsulosin, silodosin resulted in significantly better expulsion of ureteral stones <1 cm. The total complication rate did not significantly differ between silodosin and tamsulosin. Thus, silodosin may be superior to tamsulosin for MET of ureter stones <1 cm.

## 1. Introduction

The global incidence of urolithiasis, a disease with a high recurrence rate, is increasing [1]. Urolithiasis causes recurrent stone formers to experience a decline in quality of life, and there is an increasing socioeconomic burden associated with the management of urolithiasis [2]. Methods to manage ureteral stones include conservative treatment, pharmacological treatment (e.g., medical expulsive therapy (MET)), shock wave lithotripsy, and surgical treatment. Thus, urologists must select the appropriate treatment for each patient (i.e., non-surgical or surgical).

During the coronavirus disease 2019 (COVID-19) pandemic, there has been a significant change in treatment, such that emergent surgical treatment tended to decrease, whereas non-surgical treatment tended to increase [3].

According to the European Association of Urology (EAU) Guidelines on Urolithiasis, MET is recommended as a treatment option for (distal) ureteral stones >5 mm and can lessen episodes of renal colic [4]. The drugs used for MET include α-blockers, calcium channel inhibitors, and phosphodiesterase-5 inhibitors. α-blockers are regarded as the standard drugs for MET of ureteral stones, and differences in efficacy have been demonstrated among α-blockers [4]. However, whereas the American Urological Association guidelines recommend the use of α-blockers for MET of distal ureteral stones <10 mm, the guidelines do not address differences in efficacy among α-blockers [5]. Here, we performed an updated systematic review and meta-analysis of the efficacies and safety profiles of silodosin and tamsulosin, which are widely regarded as effective α-blockers for MET of ureteral stones, to explore differences in efficacy among α-blockers.

## 2. Materials and Methods

### 2.1. Inclusion Criteria

The inclusion criteria were enrollment of patients with ureteral stones; comparison of silodosin and tamsulosin for the treatment of ureteral stones; assessment of outcome measures including stone expulsion rate, stone expulsion time, and complications; and the use of a randomized controlled trial (RCT) design. The exclusion criteria were non-RCT designs; the use of α-blocker combination therapy; studies solely available in abstract, comment, or review format; and publication in languages other than English.

This report conforms to the Preferred Reporting Items for Systematic Reviews and Meta-Analyses (Supplement Table S1) [6]. Since systematic reviews and meta-analyses do not need prior approval, neither the institutional review board nor the ethics committee were required to take this study into consideration.

### 2.2. Search Strategy

A systematic review of the four English-language databases PubMed, EMBASE, the Cochrane Central Register of Controlled Trials (Central), and Web of Science was performed to identify articles published before July 2022 that described comparisons of silodosin versus tamsulosin for MET of ureteral stones. Search strategies included medical subject headings such as “urolithiasis”, “ureterolithiasis”, “ureteral calculi”, “nephrolithiasis”, “medical expulsive therapy”, “tamsulosin”, “silodosin”, and aforementioned terms.

### 2.3. Study Selection and Data Extraction

To exclude irrelevant studies, the titles and abstracts of articles discovered by the search strategy were separately evaluated by two reviewers (HDJ and DHK). From each study, the articles that were most pertinent were extracted. For included studies, the following information was recorded: author names, year of publication, country, study design, patient characteristics, treatments, and outcome variables (e.g., “stone expulsion rate”, “stone expulsion time”, and “total complication rate”).

### 2.4. Study Quality Assessment

All RCTs were subjected to risk of bias assessment using the Cochrane risk of bias tool. The qualities of the studies were independently assessed by two reviewers (HDJ and DHK) using the Scottish Intercollegiate Guidelines Network (SIGN) checklist. When two reviewers could not agree on the quality of a study, they discussed with a third reviewer (JYL).

### 2.5. Statistical Analysis

For dichotomous variables, odds ratios (ORs) and 95% confidence intervals (CIs) were calculated. For continuous variables, weighted mean differences and 95% confidence intervals were calculated. The chi-squared test (with a threshold of *p* < 0.05) was used to identify statistical heterogeneity, and the *I*^2^ statistic was used to quantify heterogeneity [7]. A fixed-effects model was used if the *I*^2^ statistic was <50%; otherwise, a random-effects model was used. The Higgins *I*^2^ statistic was calculated in the following manner:

$$I^{2}=\frac{Q-df}{Q}\times 100\%$$

where “*Q*” is the Cochrane heterogeneity statistic, and “*df*” is the degrees of freedom. Funnel plots and Egger’s test were used to assess the potential for publication bias. Additionally, sensitivity analysis was conducted to identify potential outcome reporting bias. All meta-analyses were conducted using the meta and metasens packages in R software, version 4.1.3 (R Foundation for Statistical Computing, Vienna, Austria; http://www.r-project.org (accessed on 1 September 2022)), as well as Review Manager, version 5.4.1 (RevMan, Copenhagen, Denmark: The Nordic Cochrane Center, The Cochrane Collaboration, 2020). This systematic review was registered in PROSPERO: CRD42022349671.

## 3. Results

### 3.1. Eligible Studies

**Figure 1:**
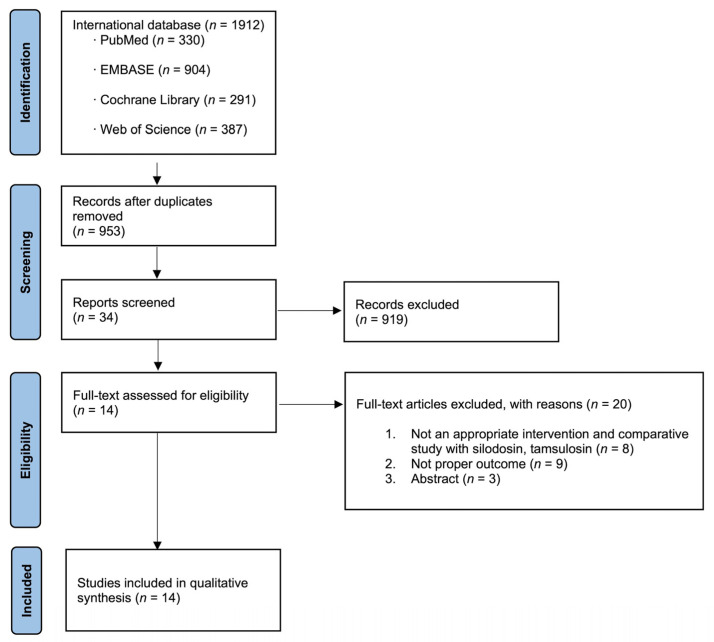
Study flow chart.

A total of 1912 studies were identified as potentially relevant. Following a full-text review, 14 RCTs and 1552 patients were chosen for inclusion in the meta-analysis (Figure 1).

### 3.2. Characteristic of Included Studies

Table 1 shows the characteristics of the 14 included studies [8,9,10,11,12,13,14,15,16,17,18,19,20,21]. These comparative studies described patients who had received silodosin and tamsulosin for MET of ureteral stones ≤1 cm. There were 11 studies of distal ureteral stones, 1 study of middle or lower ureteral stones, and 2 studies in which the location was not specified. One study comprised an RCT comparing silodosin 4 mg and tamsulosin 0.4 mg [21], while the other studies comprised RCTs comparing silodosin 8 mg and tamsulosin 0.4 mg. All included studies were RCTs published between August 2013 and July 2021.

### 3.3. Quality Assessment

**Figure 2:**
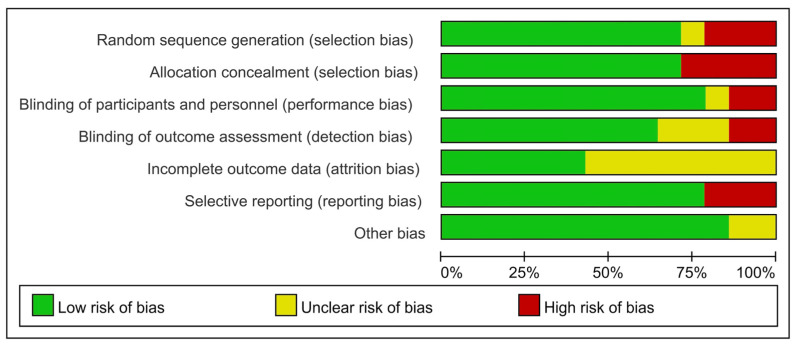
Risk of bias graph.

**Figure 3:**
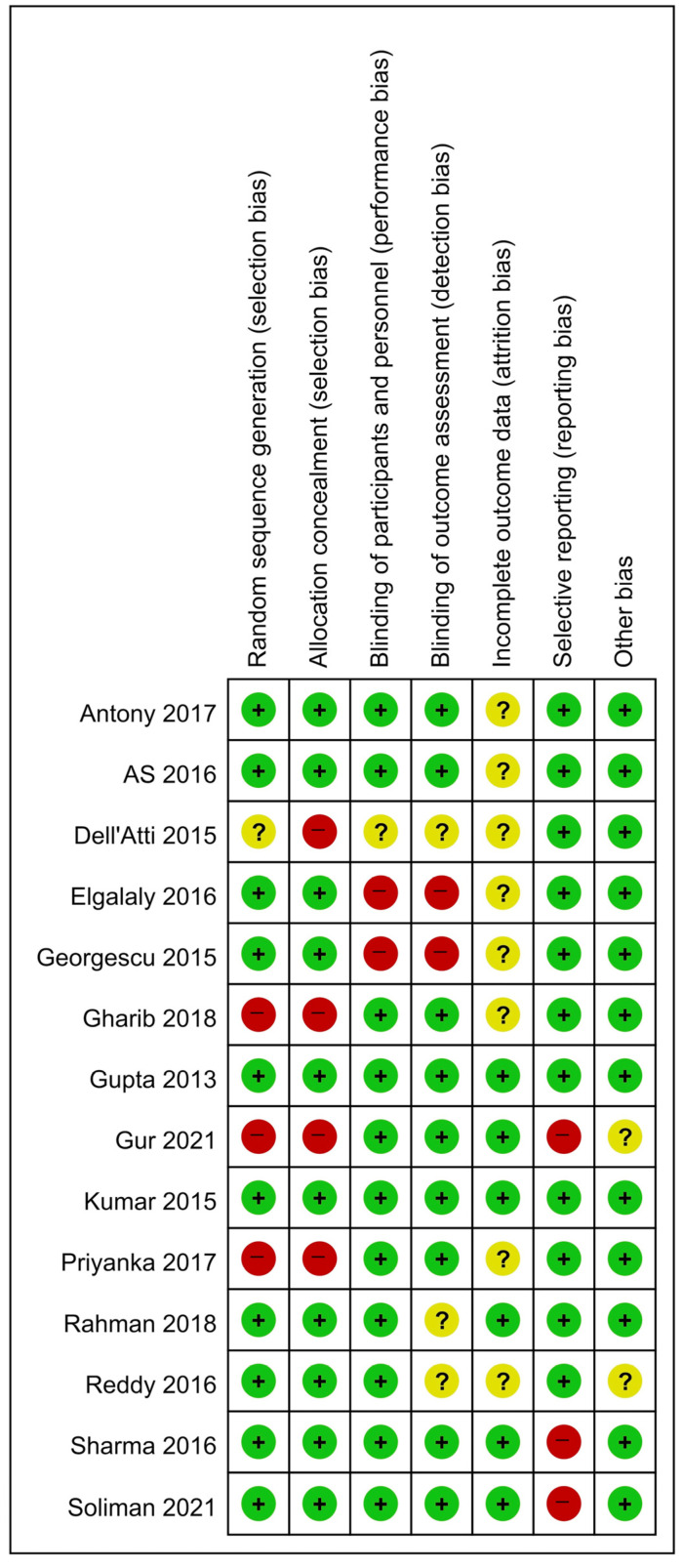
Risk of bias summary.

The qualities of the included studies were acceptable, as shown in Table 1. Five studies were rated as 1+ and the remaining nine studies were rated as 1−, according to the SIGN checklist. The risks of bias are shown in Figure 2 and Figure 3; all studies exhibited a reasonable risk of bias.

### 3.4. Publication Bias and Heterogeneity Assessment

**Figure 4:**
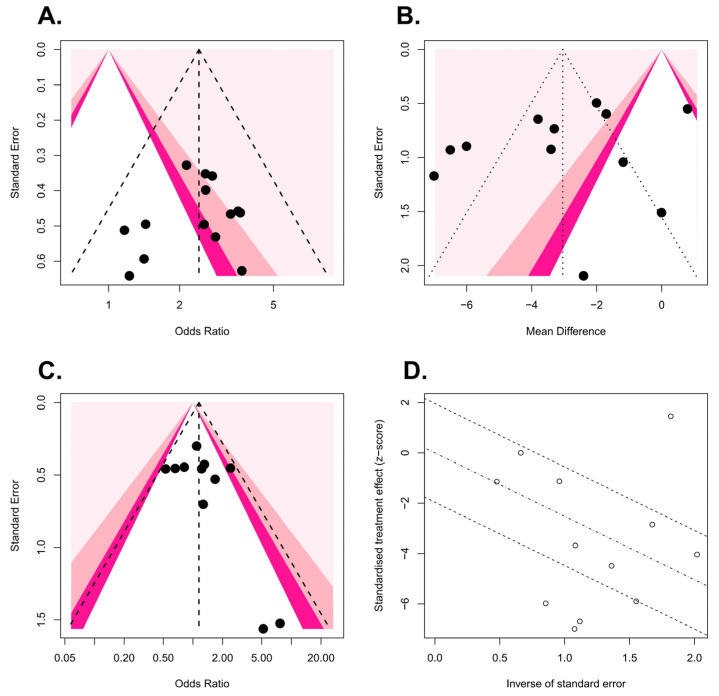
Funnel plots: (**A**) stone expulsion rate, (**B**) stone expulsion time, and (**C**) total complication rate. Radial plot: (**D**) stone expulsion time.

**Figure 5:**
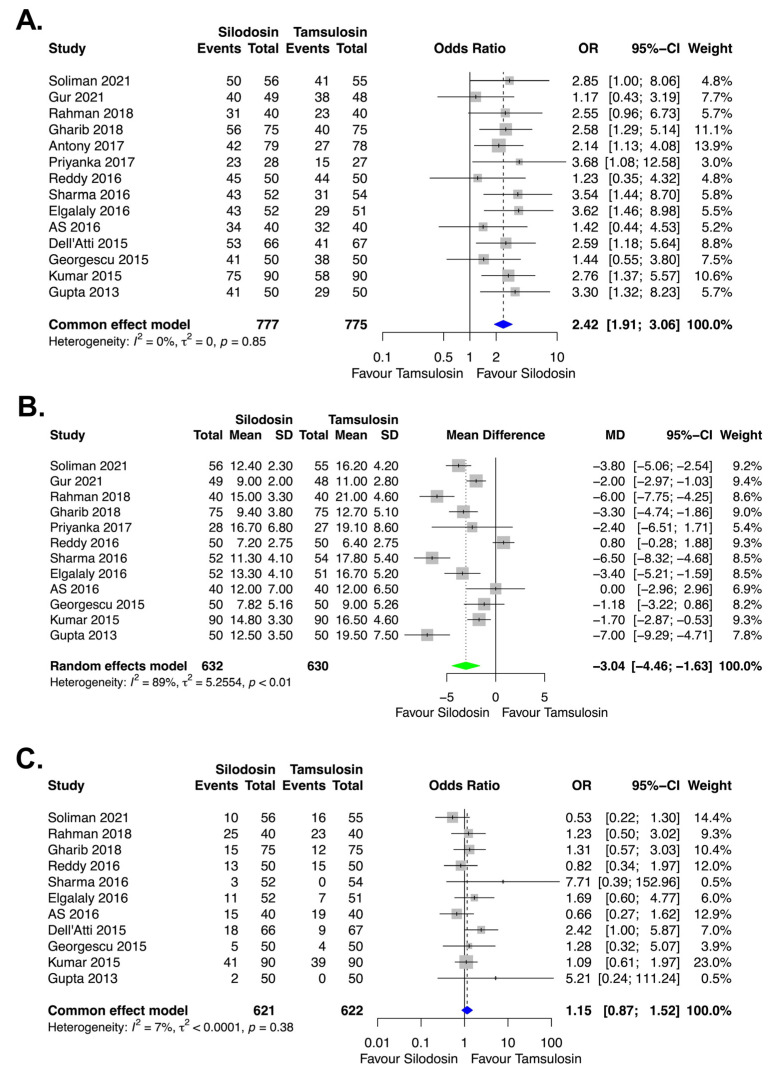
Forest plots: (**A**) stone expulsion rate, (**B**) stone expulsion time, and (**C**) total complication rate. CI, confidence interval; MD, mean difference; OR, odds ratio; SD, standard deviation.

**Figure 6:**
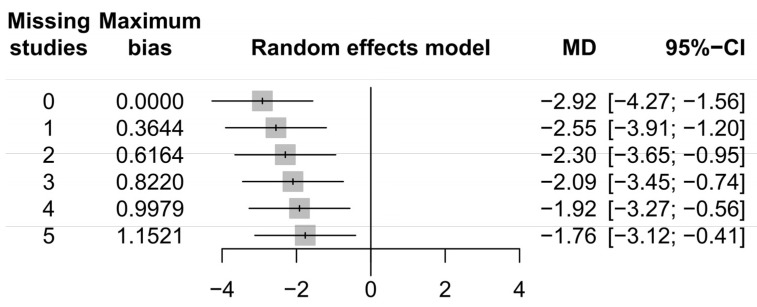
Sensitivity analysis for outcome reporting bias: stone expulsion time. CI, confidence interval; MD, mean difference.

Funnel plots of the meta-analyses are shown in Figure 4. Little publication bias was identified in Figure 4A,C, whereas some publication bias was identified in Figure 4B. However, Egger’s test showed no evidence of publication bias (Figure 4A, *p* = 0.40; Figure 4B, *p* = 0.22; Figure 4C, *p* = 0.40). Forest plots of the meta-analyses are shown in Figure 5. Little heterogeneities were observed in terms of the stone expulsion rate and total complication rate (Figure 5A, *I*^2^ = 0%; Figure 5C, *I*^2^ = 7%). Thus, fixed-effects models were used to compare the stone expulsion rate and total complication rate between silodosin and tamsulosin. High heterogeneity was observed in terms of stone expulsion time (Figure 5B, *I*^2^ = 89%). After the selection of the effect models, some heterogeneity was also observed in the radial plot of stone expulsion time (Figure 4D). Therefore, a random-effects model was used to compare stone expulsion time between silodosin and tamsulosin. Additionally, to investigate the degree of heterogeneity, a sensitivity analysis of outcome reporting bias was performed. This meta-analysis exhibited robust heterogeneity because the findings concerning stone expulsion time were not affected until at least five studies were excluded (Figure 6).

### 3.5. Stone Expulsion Rate

The stone expulsion rate was compared between silodosin and tamsulosin in 14 studies [8,9,10,11,12,13,14,15,16,17,18,19,20,21]. The maximum follow-up periods were 2 weeks in one study [11], 3 weeks in one study [13], and 4 weeks in all other studies [8,9,10,12,14,15,16,17,18,19,20,21]. Compared with tamsulosin, silodosin resulted in a significantly higher stone expulsion rate (*p* < 0.01, OR = 2.42, 95% CI = 1.91 to 3.06, *I*^2^ = 0%) (Figure 5A).

### 3.6. Stone Expulsion Time

The stone expulsion time (in days) was compared between silodosin and tamsulosin in 12 studies [8,9,10,12,14,15,16,17,18,19,20,21]. Compared with tamsulosin, silodosin resulted in a significantly shorter stone expulsion time (*p* < 0.01, mean difference (MD) = −3.04, 95% CI = −4.46 to −1.63, *I*^2^ = 89%) (Figure 5B).

### 3.7. Total Complication Rate

The total complication rate was compared between silodosin and tamsulosin in 11 studies [9,10,12,13,14,15,16,17,19,20,21]. The following complications were analyzed: orthotopic hypotension, headache, dizziness, nasal congestion, nausea, backache, retrograde ejaculation, and diarrhea. The total complication rate did not significantly differ between silodosin and tamsulosin (*p* = 0.33, OR = 1.15, 95% CI = 0.87 to 1.52, *I*^2^ = 7%) (Figure 5C).

## 4. Discussion

The COVID-19 pandemic continues to impact public health worldwide [22]. Pandemic-related shortages of hospital beds and healthcare workers have made it challenging to treat patients with common diseases [23]. Outbreaks of COVID-19 have led to hospital staffing difficulties and increased use of hospital resources for the treatment of affected patients [24]. During the Omicron variant surge, for example, the 7-day moving average of hospitals with critical staffing shortages reached nearly 22%, whereas the 7-day moving average of COVID-19 inpatient beds peaked 1–2 weeks later at approximately 21% [25].

Thus, the EAU Guidelines Office Rapid Reaction Group suggested adaptations of the EAU guidelines for use in the COVID-19 era [26]. According to these recommendations, MET and minimal interventional treatment should be used when possible to avoid surgical intervention during outbreaks of COVID-19.

Current guidelines for MET mainly recommend the use of α-blockers, rather than other drugs [4,5]. Calcium channel inhibitors (e.g., nifedipine) demonstrated efficacy in terms of stone expulsion and renal colic alleviation [27,28], but these effects were weaker than the effects of tamsulosin for distal ureteral stones [29]. Combination MET using phosphodiesterase-5 inhibitors or corticosteroids with α-blockers is not recommended because the available data were collected from studies that involved small numbers of patients [4,30].

For the first time, Borghi et al. [31] conducted an RCT of MET for ureteral stones ≤15 mm; they compared nifedipine 40 mg plus methylprednisolone 16 mg daily (group 1), with methylprednisolone 16 mg plus placebo daily (group 2). The success rate was higher in group 1 (87%, 34/39) than in group 2 (65%, 13/24). Parsons et al. [32] conducted the first meta-analysis regarding the clinical effectiveness of α-blockers for treatment of distal ureteral stones; the rate of spontaneous stone passage was 44% greater in patients receiving α-blockers (10 studies, tamsulosin 0.4 mg; 2 studies, terazosin 5 mg; 1 study, doxazosin 4 mg) than in patients who did not receive such treatment.

In a Cochrane review to compare the effectiveness of α-blockers with placebo for treatment of ureteral stones ≤10 mm, α-blockers alleviated renal colic (MD = −0.66, 95% CI: −0.91 to −0.42; *p* < 0.001; *I*^2^ = 80%) and facilitated ureteral stone expulsion (risk ratio = 1.45, 95% CI: 1.36 to 0.55; *p* < 0.001; *I*^2^ = 76%). Subgroup analysis according to α-blocker type (tamsulosin, alfuzosin, terazosin, naftopidil, or silodosin) showed no significant difference (χ^2^ = 1.44, *I*^2^ = 46.1%, *p* = 0.13) [27].

To determine the effects of specific classes of α-blockers, Yilmaz et al. [33] conducted an RCT comparing tamsulosin, terazosin, and doxazosin for MET of distal ureteral stones. Their study showed that stone expulsion rates were comparable among all three drugs.

α-blockers function through the following mechanism. The most widespread subtypes of a_1_-adrenoceptors in the distal ureter are α_1a_- and α_1d_-adrenoceptors. These α_1_-adrenoceptors are stimulated, which increases frequency and the force of ureteral contractions. However, inhibition of these receptors reduces basal ureteral tone, as well as peristaltic frequency and amplitude; these changes reduce intraluminal pressure while enhancing the rate of urine transport, thereby increasing the likelihood of stone passage [34].

Itoh et al. [35] reported that, in the human ureter, the expression of α_1d_-adrenoceptors was greater than the expression of α_1a_-adrenoceptors. Additionally, Tomiyama et al. [36] investigated α_1_-adrenoceptor subtypes in the hamster ureter according to gene and protein expression patterns, as well as contractile function. They found that α_1d_-adrenoceptors were more prevalent than α_1a_-adrenoceptors, but ureteral smooth muscle contraction was mainly mediated by α_1a_-adrenoceptors. Subsequently, Sasaki et al. [37] reported that α_1a_-adrenoceptors were the main receptors involved in phenylephrine-induced contraction in the human ureter, suggesting that α_1a_-adrenoceptors have a central role in contraction in the human ureter.

Thus, Itoh et al. [38] conducted the first prospective RCT of silodosin for MET of ureteral stones <10 mm. Compared with patients instructed to consume 2 L of water daily, patients who received silodosin 8 mg daily exhibited a shorter mean stone expulsion time and higher stone expulsion rate. Since that RCT of silodosin, meta-analyses have shown that silodosin is more potent than tamsulosin for MET of ureteral stones [39,40]. In a meta-analysis with five RCTs, Liu et al. [40] found that silodosin significantly increased the expulsion rate of distal ureteral stones, compared with tamsulosin, but there was no significant difference between treatments in terms of stone expulsion time or retrograde ejaculation rate. Hsu et al. [39] conducted a meta-analysis with 13 RCTs (including 2 RCTs solely available in abstract format) and 3 observational studies. They found that, compared with tamsulosin, silodosin resulted in a higher stone expulsion rate and shorter expulsion time; however, silodosin also led to a higher incidence of retrograde ejaculation.

Here, we conducted an updated systematic review and meta-analysis of 14 RCTs to compare the efficacies and safety profiles of silodosin and tamsulosin for MET of ureteral stones ≤1 cm. We found that, compared with tamsulosin, silodosin resulted in a higher stone expulsion rate and shorter stone expulsion time. Moreover, we found no difference in the total complication rate between silodosin and tamsulosin.

Experimental studies have demonstrated that α_1a_-adrenoceptors are the main receptors involved in contraction in the human ureter. Furthermore, the results of previous meta-analyses and our meta-analysis suggest that silodosin is superior to tamsulosin for MET of ureteral stones.

This updated systematic review and meta-analysis had some limitations. First, one of the analyzed studies comprised an RCT conducted with silodosin 4 mg [21], whereas other studies were RCTs conducted with silodosin 8 mg. Despite the lower dose of silodosin, the study involving silodosin 4 mg showed that the stone expulsion rate was higher and the stone expulsion time was shorter with silodosin than with tamsulosin. Silodosin 4 mg also performed better than tamsulosin in terms of the total complication rate, which might have influenced the meta-analysis finding of no significant difference in the total complication rate. Second, we could not conduct subgroup analysis according to ureteral stone location because 11 studies focused on distal ureteral stones, 1 study focused on middle or lower ureteral stones, and 2 studies focused on an unspecified location. Third, we could not conduct subgroup analysis by dividing ureteral stones according to size (e.g., ≤5 mm and 5–10 mm). Although five studies had inclusion criteria of ureteral stone size 5–10 mm, the remaining nine studies had inclusion criteria of ureteral stone size <10 mm and did not separately evaluate ureteral stones ≤5 mm.

## 5. Conclusions

This updated systematic review and meta-analysis of RCTs showed that, compared with tamsulosin, silodosin performed significantly better in the expulsion of ureteral stones <1 cm. The total complication rate did not differ between silodosin and tamsulosin. Thus, silodosin may be preferable to tamsulosin for MET of ureteral stones <1 cm.

## Figures and Tables

**Table 1:** Characteristics of included studies.

Study	Design	Medication	No. of Patients	Age: Mean (SD), Years	Stone Size: Mean (SD), mm	Inclusion Criteria	Quality Assessment
Soliman et al. 2021 [21]	RCT	Silodosin 4 mg	56	11.1 (1.8)	6.2 (1.2)	Distal ureteral stone <1 cm	1+
		Tamsulosin 0.4 mg	55	11.4 (2.4)	6.3 (0.9)		
		Placebo	56	11.2 (2.6)	6.5 (1)		
Gur et al. 2021 [18]	RCT	Dexketoprofen 50 mg	50	38 (31.5–48)	5.75 (4.77–8.12)	Distal ureteral stone 4–9.9 mm	1−
		Tamsulosin 0.4 mg	48	41 (30.25–51.75)	6.2 (4.62–7.67)		
		Silodosin 8 mg	49	41 (33–0.5)	6.2 (5.1–7.55)		
		Tadalafil 5 mg	46	39 (31.75–48)	6.1 (5.17–7.37)		
Rahman et al. 2018 [20]	RCT	Tamsulosin 0.4 mg	40	38 (10)	7.5 (1.2)	Distal ureteral stone 5–10 mm	1+
		Silodosin 8 mg	40	34 (12)	7.4 (1.3)		
		Silodosin 8 mg and tadalafil 5 mg	40	35 (10)	7.6 (1.35)		
Gharib et al. 2018 [16]	RCT	Silodosin 8 mg	75	34.5 (9.8)	7.47 (1.41)	Lower third of the ureter 5–10 mm	1−
		Tamsulosin 0.4 mg	75	34.8 (9.7)	7.54 (4.3)		
Antony et al. 2017 [11]	RCT	Silodosin 8 mg	79	NA	NA	Ureteral stone <10 mm	1+
		Tamsulosin 0.4 mg	78	NA	NA		
Priyanka et al. 2017 [8].	RCT	Tamsulosin 0.4 mg	27	34.8 (12.7)	NA	Distal ureteral stones <10 mm	1−
		Silodosin 8 mg	28	36.4 (12.7)	NA		
Reddy et al. 2016 [9]	RCT	Tamsulosin 0.4 mg	50	39.4 (21–70)	NA	Lower ureteral stone <10 mm	1−
		Silodosin 8 mg	50	38.2 (21–70)	NA		
Sharma et al. 2016 [10]	RCT	Silodosin 8 mg	52	NA	NA	Distal ureteral stone <10 mm	1−
		Tamsulosin 0.4 mg	54	NA	NA		
Elgalaly et al. 2016 [14]	RCT	Silodosin 8 mg	52	33.6 (9.9)	5.4 (1.5)	Distal ureteral stones <10 mm	1−
		Tamsulosin 0.4 mg	51	35.5 (11.3)	5.6 (1.2)		
AS et al. 2016 [12]	RCT	Tamsulosin 0.4 mg	40	35 (8.5)	7 (2)	Distal ureteral stones <10 mm	1−
		Silodosin 8 mg	40	32 (7.5)	7 (1.5)		
		Control (diclofenac 50 mg to 100 mg prn)	40	34 (8.5)	6.8 (1.8)		
Dell’Atti et al. 2015 [13]	RCT	Tamsulosin 0.4 mg	67	35 (21–64)	5.37 (1.33)	Lower ureteral stone 4–10 mm	1−
		Silodosin 8 mg	66	36 (19–72)	5.82 (1.66)		
Georgescu et al. 2015 [15]	RCT	Tamsulosin 0.4 mg and diclofenac 50 mg	50	43.5 (13.31)	5.08 (2.09)	Ureteral stone <10 mm	1−
		Silodosin 8 mg and diclofenac 50 mg	50	44.26 (13)	5.32 (2.09)		
		Anti-inflammatory drugs	50	45.14 (11.58)	5.1 (2.02)		
Kumar et al. 2015 [19]	RCT	Tamsulosin 0.4 mg	90	36.4 (10.03)	7.44 (1.2)	Distal ureteral stones 5–10 mm	1+
		Silodosin 8 mg	90	36.73 (12)	7.5 (1.3)		
		Tadalafil 10 mg	90	37.5 (13.5)	7.77 (1.35)		
Gupta et al. 2013 [17]	RCT	Tamsulosin 0.4 mg	50	NA	7 (2.3)	Middle or lower ureteral stones <1 cm	1+
		Silodosin 8 mg	50	NA	6.6 (1.8)		

RCT, randomized controlled trial; SD, standard deviation. The quality assessment was indicated by the Scottish Intercollegiate Guidelines Network checklist, whereby: 1+ means well-conducted RCT with a low risk of bias, 1− means RCT with a high risk of bias, 2+ means well-conducted cohort studies with a low risk of bias, 2− indicates cohort studies with a high risk of bias.

## Data Availability

The data presented in this study are available in the article.

## Supplementary Materials

The following supporting information can be downloaded at: https://www.mdpi.com/1648-9144/58/12/1794/s1, Table S1: PRISMA Checklist.
